# Can chlorhexidine gluconate baths reduce fungal colonisation in intensive care unit patients?

**DOI:** 10.1186/s13756-025-01606-6

**Published:** 2025-07-09

**Authors:** Teresa Nascimento, João Inácio, Daniela Guerreiro, Patrícia Patrício, Luís Proença, Cristina Toscano, Priscila Diaz, Helena Barroso

**Affiliations:** 1 School of Health & Science, Egas Moniz Center for Interdisciplinary Research (CiiEM), Egas Moniz, Caparica, Almada, 2829-511 Portugal; 2 School of Applied Sciences, University of Brighton, Brighton, UK; 3 Hospital da Luz, Setúbal, Portugal; 4 Centro Hospitalar Lisboa Ocidental Hospital Egas Moniz, Lisboa, Portugal; 5 Hospital Prof. Doutor Fernando da Fonseca, Amadora, Portugal

**Keywords:** Chlorhexidine gluconate, *Candida* spp., Intensive care unit, Colonisation; infection prevention

## Abstract

**Background:**

Chlorhexidine Gluconate (CHG) bathing is widely used in Intensive Care Units (ICUs) to reduce bacterial colonisation, yet its efficacy against fungal skin colonisation, particularly *Candida* spp., is not well understood. This study aimed to evaluate the impact of daily CHG bathing on *Candida* colonisation among ICU patients.

**Methods:**

From 2020 to 2022, axillary/inguinal swabs were collected from 675 ICU patients across three units on admission (Day 1, D1), Day 5 (D5) and Day 8 (D8). Patients received daily CHG bathing (either 2% impregnated wipes or 4% liquid solution) from D1 to D5, followed by soap-and-water bathing from Day 6 to D8. Standard and molecular microbiological methods were used to identify fungal species, and colony-forming units (CFUs) were quantified. Colonisation rates and fungal burden were compared across time points and bathing protocols.

**Results:**

A total of 988 swabs from 675 patients were collected, 675 on D1, 203 on D5 and 110 on D8. CHG bathing had no significant impact on *Candida* burden at individual time points, (D1, *p* = 0.223; D5, *p* = 0.939 and D8, *p* = 0.669). No significant differences in colonisation or fungal burden were observed between the use of 4% CHG solution and 2% CHG-impregnated wipes upon ICU admission. However, in the subgroup of 89 patients monitored longitudinally, a transient reduction in colonisation was observed during the CHG bathing period (D1–D5), followed by a significant increase during the soap-and-water period (D6–D8) (*p* = 0.005; between periods: *p* < 0.001). Among the 329 positive swabs, 274 yielded > 100 CFU/ml. High colony counts of *C. albicans* (> 1000 CFU/mL) were observed, with no significant association between colonisation levels and specific *Candida* species (*p* = 0.940).

**Conclusions:**

CHG bathing demonstrated only a limited and transient impact on *Candida* colonisation in ICU patients. Colonisation rates rebounded after cessation of CHG use, suggesting ongoing acquisition during ICU stay. These findings highlight the need for additional or alternative infection control measures targeting fungal pathogens in critical care settings.

## Background

In the Intensive Care Unit (ICU), patients face a significantly heightened risk for fungal healthcare-associated infections (HAIs) due to a variety of contributing factors. These include the frequent use of invasive procedures, the presence of a weakened immune system, and the need for prolonged hospital stays [1, 2]. Therefore, infection control within ICUs is of paramount importance due to the high vulnerability of patients to various nosocomial infections, including those caused by multidrug-resistant organisms (MDROs), including methicillin-resistant *Staphylococcus aureus* (MRSA), vancomycin-resistant *Enterococci* (VREs), *Acinetobacter baumannii*, carbapenemase-producing *Enterobacteriaceae* (CPEs), *Pseudomonas aeruginosa*, and *Candida* spp. [3]. Among *Candida* species, emerging multidrug-resistant *Candidozyma auris* (*Candida auris)* and azoles resistant *Candida parapsilosis* stand out. These fungal pathogens are particularly concerning because of their ability to colonise the skin and subsequently cause severe infections, posing substantial challenges to patient management and infection control efforts [4, 5].

Compared with infections caused by susceptible microorganisms, infections caused by MDROs in the ICU have a worse prognosis, necessitating heightened concern and increased prevention efforts [6]. Comprehensive infection control strategies encompass hand hygiene, environmental cleaning and disinfection, isolation measures, patient decolonisation, and antimicrobial resistance prevention programs [7]. The effectiveness of these measures depends on the specific microorganism, adherence to protocols, and clinical context.

One of the critical strategies employed to mitigate these risks is the routine use of Chlorhexidine Gluconate (CHG) baths. CHG, an antiseptic agent with broad-spectrum antimicrobial activity, has been widely adopted in ICU settings for its proven efficacy in reducing bacterial colonisation and transmission of MDROs [8, 9].

CHG can be applied in several forms, each with its own specific concentration and method of use. The two primary forms are liquid CHG solutions and CHG-impregnated wipes. Liquid CHG solutions are commonly available at concentrations ranging from 2% (v/v) to 4% (v/v) and can be used as topical antiseptics for washing or bathing [8]. Alternatively, CHG-impregnated wipes offer a convenient and standardized method of application, typically containing a 2% (v/v) concentration of CHG. These wipes are premoistened and ready for immediate use, making them efficient options for busy clinical environments [10].

*Candida* colonisation during CHG bathing has been understudied due to a historical focus on bacterial pathogens, limited recognition of fungal significance, and diagnostic challenges. Research priorities often centred on CHG’s antibacterial effects, with fungal issues gaining attention only recently due to rising antifungal resistance and vulnerable patient populations [5].

While the primary focus of these studies has been on MDROs, there are indications that CHG bathing may also be beneficial in reducing *Candida* colonisation, namely *C. auris* [11]. CHG is cationic and relies on interactions with negatively charged surfaces. Bacteria often have negatively charged surfaces due to teichoic acids in Gram-positive bacteria and lipopolysaccharides in Gram-negative bacteria [8]. The fungal cell wall is more complex, consisting of layers of chitin, glucans (primarily β-1,3-glucans), and mannoproteins. Theoretically based, the less negatively charged and hydrophobic nature of fungal cell walls reduces CHG’s ability to bind and disrupt the wall effectively. Additionally, *Candida* species frequently form biofilms, which significantly impairs CHG penetration and antimicrobial action [5].

CHG in in vitro studies has shown potent antifungal activity against *C. auris* in controlled laboratory settings. Johnson et al. (2021) reported that CHG effectively inhibited *C. auris* growth, highlighting its potential as a preventive agent [12]. Clinical studies have indicated variable outcomes, with some demonstrating reduced colonisation of *Candida* species on the skin following CHG bathing. However, effectiveness can depend on factors like skin condition, application protocols, and the strain of *Candida* [13].

This potential advantage suggests that further research specifically targeting the effects of CHG on *Candida* is warranted to fully understand and optimize its use in clinical practice.

## Methods

### Aim, Study design

We aimed to evaluate the burden of *Candida* colonisation in ICU patients during routine CHG bathing to better understand and optimize its use in clinical practice. More specifically, to determine the rates of *Candida* colonisation in ICU patients at different time points.

This prospective multicentre study was conducted from January 2020 to December 2022, in suburban Lisbon, at two tertiary large hospitals belonging to the Portuguese National Public Health Service: *Hospital Professor Doutor Fernando Fonseca* (FFH), an 802-bed hospital with two ICUs (general and surgery) and *Beatriz Ângelo Hospital* (BAH), a 424-bed hospital with one general ICU. Participation in the study was voluntary and authorized by signing an informed consent form. All patients under the age of 18, pregnant women, and mentally disabled individuals were not included in the study.

Drawing on prior studies of fungal colonisation in ICUs, the required sample size was estimated at 865 patients to achieve a 95% confidence level and 80% statistical power [14–18].

The sampling from each patient was performed by a non-invasive, bilateral axillary/inguinal combined swab. Samples were collected when patients were admitted to the ICU (D1) and continued throughout their ICU stay, specifically on the 5th day (D5) and the 8th day (D8), depending on the length of their stay in the ICU.

The infection control protocols differed between the two hospitals. At FFH, each patient received a bath with wipes saturated in a 2% (v/v) chlorhexidine gluconate (CHG), while BAH employed a CHG protocol using a different concentration and form, namely a 4% (v/v) CHG liquid soap for the same period. All patients received a CHG bath before swab collection. CHG baths were administered daily from D1 through D5, followed by soap-and-water bathing from Day 6 (D6) to D8. Patients received their initial CHG bath on the day of ICU admission, regardless of the time of admission. CHG or soap-and-water bathing protocols were implemented consistently across each unit as part of standard operating procedures. All CHG baths were administered by ICU nursing staff as part of standard clinical care; neither patients nor members of the study team were involved in product application. Bathing compliance was recorded by auxiliary staff in accordance with each hospital’s quality assurance program, which includes strict adherence to standardized infection control procedures and protocol documentation, and based on these routine records, reported compliance was consistently close to 100%.

### Microbiological protocol

**Fig. 1:**
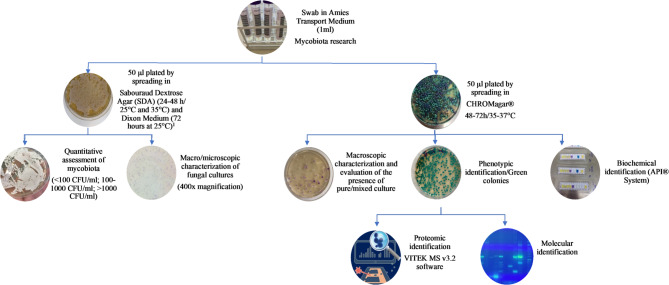
Protocol for mycological screening and determination of colonisation density of mycobiota in intensive care units. ^1^Inoculation in Dixon Medium was only carried out for samples from the FFH ICUs

Swabs were collected in a Σ-transwab^®^ system transport (Sigma Transwab- Liquid Amies), transported at room temperature (25 °C) and processed within 24–48 h. Each swab containing 1 ml of liquid Amies medium was subjected to strong mechanical agitation for 30 s and 50 µl aliquots were inoculated by spreading onto the following culture media: Sabouraud Gentamicin Chloramphenicol 2 agar (SGC2) (bioMérieux, Marcy l’Etoile, France) and a commercial chromogenic medium for *Candida*, CHROMagar Candida^®^ (CHROMagar, Paris, France). CHROMagar Candida^®^ has a mixture of chromogenic substrates, each of which is specific for enzymes of the most common *Candida* species. Thus, the colour developed by yeasts of *Candida* spp. grown on CHROMagar Candida^®^ medium shows green colonies for *C. albicans*; metallic blue colonies for *C. tropicalis*; light pink and dry colonies for *C. krusei* and white to violet colonies for the other *Candida* species. According to the manufacturer, the specificity and sensitivity for *C. tropicalis*, *C. albicans* and *C. krusei* exceed 99%. In addition to direct presumptive identification, this culture medium allowed mixed cultures to be identified. The plates were incubated aerobically. For the SGC2 plates, one set was incubated at 25 °C and another at 35 °C for 24–48 h. The CHROMagar Candida plates were incubated at a temperature range of 35–37 °C for 48–72 h. Dixon’s medium was used to highlight all the species of *Malassezia*. The plates were incubated aerobically for 72 h at 25 °C (Fig. 1).

### Species identification

The identification of the isolates was based on the standard criteria of their macroscopic and microscopic morphology, growth temperature, biochemical profile of aerobic sugar assimilation and appearance on chromogenic agar (Fig. 1). All the isolates were further processed for analysis with MALDI-TOF MS—VITEK MS (bioMérieux, Marcy l’Etoile, France) using VITEK MS v3.2 software [19]. All identifications displaying a single result with a confidence value of 99.9% were considered acceptable (Fig. 1).

Molecular methods were employed to analyse the three *Candida* species complexes (*C. albicans*, *C. glabrata*, and *C. parapsilosis*) most closely linked to human infections. This approach aimed to identify potential cryptic species within these complexes [20–22]. The protocol, optimized from the one developed by Arastehfar et al., was designed to simultaneously identify cryptic species within the *C. albicans*, *C. glabrata*, and *C. parapsilosis* complexes [23]. Additionally, all *Candida* isolates were subjected to a polymerase chain reaction (PCR) assay specific for *C. auris* [24]. For this purpose, total DNA was extracted from the isolates using the NZYMicrobial gDNA Isolation Kit^®^ (Nzytech, Lisboa, Portugal), following the manufacturer’s instructions. The PCRs were performed using a T100 thermal cycler (Bio-Rad Laboratories, Hercules, California, USA). The amplified products were analysed on 2% agarose gels stained with greensafe (Nzytech, Lisboa, Portugal) and visualized using a UV transilluminator (Fig. 1).

### Quantification of growth

After incubation, the total number of yeast colonies was quantified based on the initial volume spread and defined as the relative intensity of colonisation. The total number of viable microorganisms was counted on SGC2 and expressed as Colony Forming Units per millilitre (CFU/ml). To ensure practical and accurate assessment, the counts were categorized into three groups: less than 100 CFU/ml, 100–1000 CFU/ml, and more than 1000 CFU/ml. Specifically, counts below 100 CFU/ml were recorded as a maximum of 5 CFU, counts between 100 and 1000 CFU/ml as a maximum of 50 CFUs, and counts exceeding 1000 CFU/ml as more than 50 CFUs. An increased colonisation burden was defined as the detection of more than 50 CFUs. Cultures were visually examined at 24 and 48 h, with evaluations independently conducted by two qualified investigators.

### Statistical analysis

To describe the demographic, clinical, and mycological characteristics of the study population, data were collected and analysed using the IBM Statistical Package for the Social Sciences (SPSS) for Windows version 29.0 (IBM Corp., Armonk, NY). An exploratory and descriptive analysis was conducted to identify patterns within each variable in the sample. Categorical variables are presented as frequencies and percentages. For inferential analyses, a *p*-value of less than 0.05 was considered statistically significant.

## Results

### Patient characteristics

**Fig. 2:**
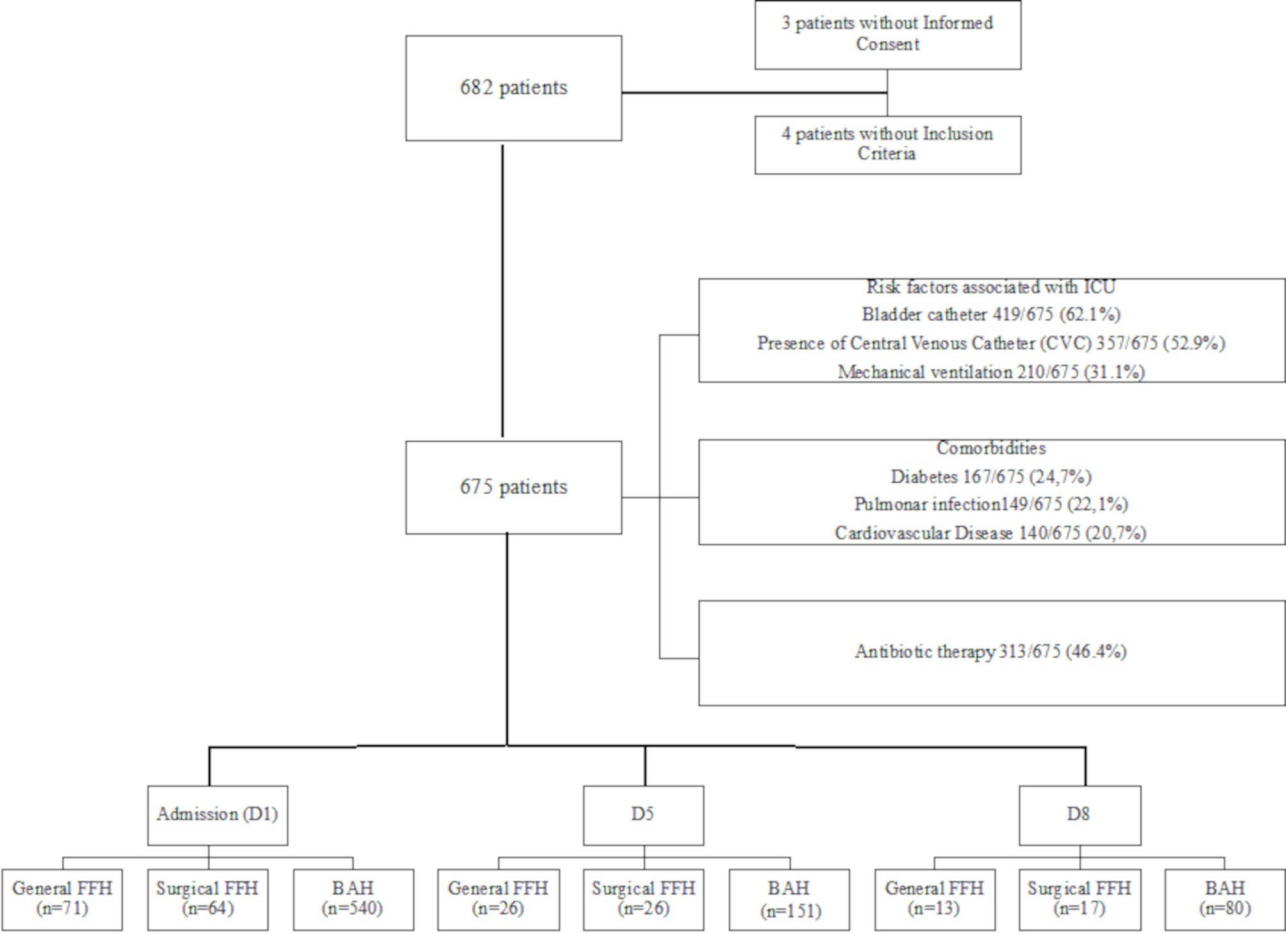
Flowchart of the prospective study population cohort

The study enrolled 675 patients over two years, excluding three who refused consent and four who were under 18. Participants included 64 from the surgical ICU, 71 from the general ICU at FFH (pre-COVID, Jan-Mar 2020), and 540 from the BAH ICU (from Sept 2021) (Fig. 2). A total of 988 swabs were collected from 675 patients, with each patient contributing one or more swabs depending on the timepoints during their ICU stay. Of the patients sampled on D1, 203 had follow-up collections on D5. On D8, samples were obtained from 110 patients, of whom 89 had sequential collections on Days 1, 5, and 8, while 21 patients had only D1 and D8 samples without an intermediate D5 collection.

In the cohort under study, 401 patients (60.0%) were male, and 566 (83.0%) were caucasian, with a mean age of 67 years. The majority of patients had a central venous catheter (CVC) (52.9%; 357/675), almost half had received previous antibiotic therapy (46.4%; 313/675) and (31.1%; 210/675) were on mechanical ventilation (Fig. 2). Detailed information on the epidemiology and predisposing risk factors for *Candida* spp. colonisation has been comprehensively described in a previous publication of our investigation [25].

### Colonisation rates

**Fig. 3:**
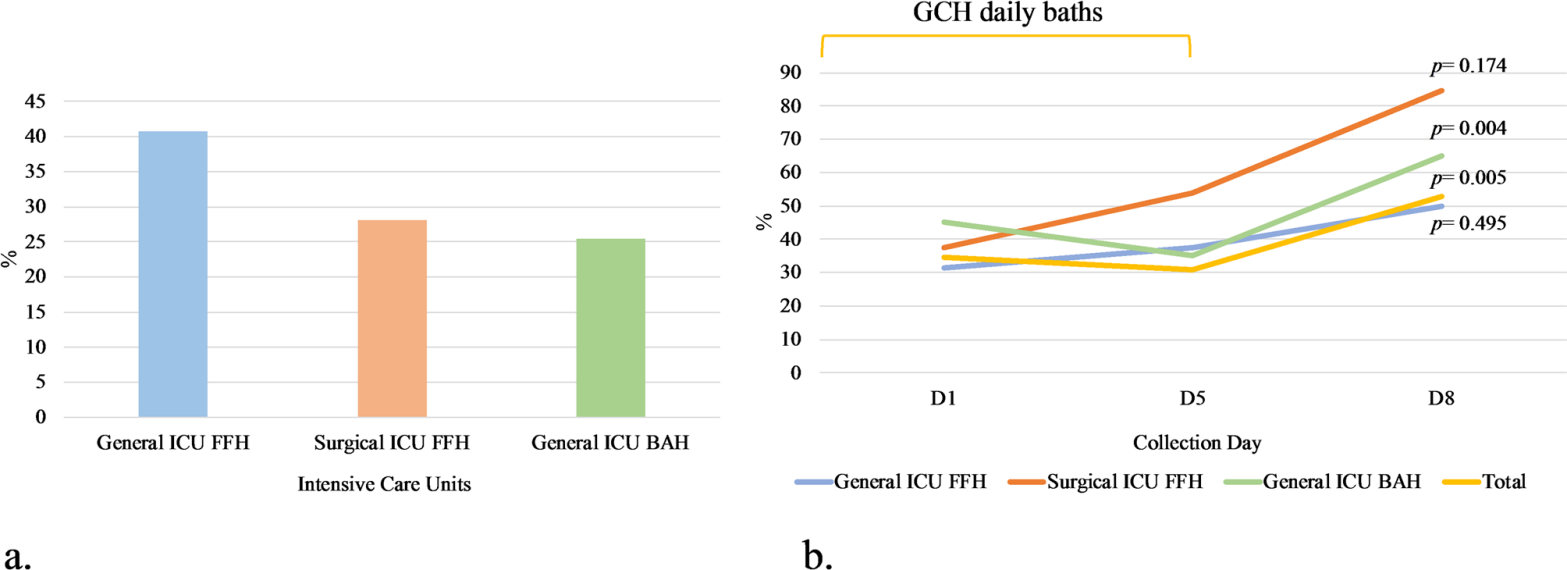
(**a**) Overall distribution of patients colonised by fungi across hospital units. (**b**) Fungal colonisation profile throughout the entire length of stay in the General ICU-FFH, Surgical ICU-FFH and General ICU- BAH. In the General ICU-FFH unit, colonisation rates increased from 31.3% on Day 1 (D1) to 37.5% on Day 5 (D5), reaching 50.0% by Day 8 (D8). In the Surgical ICU-FFH unit, a more pronounced rise was observed, from 37.5% on D1 to 53.8% on D5 and 84.6% on D8. Conversely, in the BAH unit, colonisation was initially higher at 45.0% on D1 but decreased to 35.0% on D5, before increasing again to 65.0% on D8.

Among all patients, 232 (34.4%) were colonised by yeast species during their ICU stay. Of these, 15.3% acquired colonisation during hospitalization, and 29.2% remained persistently colonised across the observation period. Colonisation upon admission was highest in the General ICU at FFH (40.8%; 29/71 patients), followed by the Surgical ICU at FFH (28.1%; 18/64 patients) and the BAH ICU (25.4%; 137/540 patients), with the difference reaching statistical significance (*p* = 0.007) (Fig. 3a).

Overall, colonization was 184/675 (27.3%), 87/203 (42.8%), and 58/110 (52.7%) on D1, D5, and D8, respectively [25]. When comparing the CHG bathing period (D1–D5) to the soap-and-water period (D6–D8), a statistically significant difference in colonisation rates was found (*p* < 0.001), with 82.4% of colonisation events occurring during CHG use and 17.6% afterward. Longitudinal follow-up of 89 patients showed an overall increase in colonisation rates over time, with a temporary decrease observed on Day 5 (Fig. 3b). Each ICU exhibited distinct colonisation trends throughout the study period. In the General ICU at FFH, colonisation rates exhibited a gradual increase, rising from 31.3% on Day 1 (D1) to 37.5% on Day 5 (D5), and reaching 50.0% by Day 8 (D8), though this change was not statistically significant (*p* = 0.495). A more marked increase was seen in the Surgical ICU at FFH, where colonisation rose from 37.5% on D1 to 53.8% on D5, peaking at 84.6% on D8 (*p* = 0.174). In contrast, the General ICU at BAH showed a different trend: colonisation began at a higher baseline of 45.0% on D1, decreased to 35.0% on D5, but then sharply increased to 65.0% on D8. This change was statistically significant (*p* = 0.004), suggesting a potential rebound in colonisation following the cessation of CHG bathing. When aggregating data across all ICUs, the overall colonisation rate showed a modest decline from 34.5% on D1 to 30.9% on D5, followed by a significant increase to 52.7% on D8 (*p* = 0.005) (Fig. 3b). To further explore whether these trends were influenced by the CHG daily baths, subgroup analysis was conducted and significant differences in fungal colonisation were observed between the CHG bathing (D1–D5) and soap-and-water periods (D6–D8) (*p* < 0.001). These findings underscore the dynamic nature of *Candida* colonisation during ICU stay and suggest a temporal association between bathing protocols and colonisation trends.

### Fungal colonisation rates by CHG formulation

A subgroup analysis was performed to assess whether the type or formulation of CHG used influenced fungal colonisation. Two ICUs applied CHG using 2% (v/v) CHG-impregnated wipes, while a third ICU used 4% (v/v) CHG liquid soap. Univariate analysis from Day 1 to Day 8 showed no statistically significant differences in colonisation rates among these groups. The colonisation rate was 30.8% in the general ICU using 2% CHG wipes (OR 1.12, *p* = 0.860), 31.3% in the surgical ICU using the same wipes (OR 1.15, *p* = 0.819), and 28.3% in the general ICU using 4% CHG soap (reference group, OR 1.00) (Table 1).

**Table 1:** Comparison of fungal colonisation rates by CHG formulation and ICU type (Day 1–Day 8)

ICU Type	CHG Formulation	Colonisation Rate (D1–D8)	Odds Ratio (OR) (95% CI)	*p*-value
General ICU	2% CHG wipes	30.8%	1.12 (0.30–4.14)	0.860
Surgical ICU	2% CHG wipes	31.3%	1.15 (0.35–3.81)	0.819
General ICU	4% CHG liquid soap	28.3%	1	–

Our findings did not demonstrate any evidence of differences in fungal colonisation reduction between the 4% CHG solution and the 2% CHG-impregnated wipes upon ICU admission.

### Fungal diversity

Fungal species diversity was analyzed based on a total of 371 fungal isolates obtained from 329 culture-positive samples. While most samples yielded a single isolate (286/329, 86.9%), 43 samples (13.1%) yielded mixed cultures with two or more fungal species present. We identified four genera of yeast-like fungi with a predominance of the genus *Candida* (95.7%, 355 out of 371), predominantly *C. albicans* and *C. parapsilosis* complex, followed by species of the genus *Rhodotorula* (2.4%, 9 out of 371), *Trichosporon* (1.6%, 6 out of 371) and *Saccharomyces* (0.3%, 1 out of 371).

Among the samples that presented mixed cultures, 13.1% (43/329) were composed mainly of *C. albicans* and one non-*albicans* species (NAC) (86%; 37/43). Mixed cultures were particularly observed in D1, with a decrease over the collection period [D1 (58.1%; 25/43); D5 (27.9%; 12/43) and D8 (14.0%; 6/43)].

### Colony counts

**Fig. 4:**
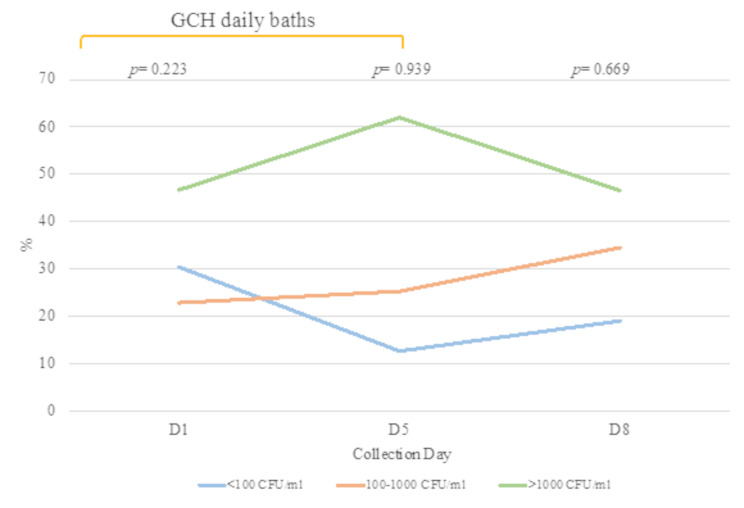
Total colony counts recorded across all collection days in the ICU

Among the positive samples, 50.8%, 25.4% and 22.8% presented fungal growth above 1000 CFU/mL, average densities of 100–1000 CFU/mL and below 100 CFU/mL, respectively. Some variations in the number of CFU/mL could be observed throughout the ICU stay. For samples with < 100 CFU/mL, there was a slight decrease after admission to the ICU and an increase after D5 of hospitalisation (D1: 30.4%; D5: 12.6% and D8: 19.0%). For colony counts between 100 and 1000 CFU/mL, there was a linear increase across the prevalence points (D1: 22.8%; D5: 25.3%; D8: 34.5%). The density of the colonised samples (> 1000 CFU/mL) gradually increased until D5 of hospitalisation and by D8, they showed colonisation rates similar to those of D1 (D1: 46.7%; D5: 62.1%; D8: 46.6%) (Fig. 4). However, no significant differences were detected at any of the collection points or in the intensity of CFU/mL (D1, *p* = 0.223; D5, *p* = 0.939 and D8, *p* = 0.669). Overall, these trends that while high fungal loads predominated overall, their distribution varied temporally, with moderate-burden samples progressively increasing and low-burden samples showing a transient dip during mid-stay.

For *Candida* species, an additional analysis was performed to explore potential associations between the identified *Candida* species and the fungal burden (CFU/mL) at each collection point during ICU stay. The colony counts of *C. albicans* were high, exceeding 1000 CFU/mL. However, there was no statistically significant association between the degree of colonisation and the specific *Candida* species (*p* = 0.940). Considering all collection points, a significant reduction in colonisation density was observed for *C. glabrata* on D8, with levels significantly decreasing below 100 CFU/mL (*p* = 0.048) (Table 2).

**Table 2:** Distribution of CFUs by *Candida* species and day of collection (D1, D5 and D8)^1^

*Candida* spp. *n* = 355	Total	< 100 CFU/ml *n*= 81	100–1000 CFU/ml *n*= 90	> 1000 CFU/ml *n*= 184	*p*
*C. albicans*	185/355 (52.1)	39/81 (48.1)	46/90 (51.1)	100/184 (54.3)	0.633
D1	95/196 (48.5)	27/56 (48.2)	19/45 (42.2)	49/95 (51.6)	0.585
D5	53/96 (55.2)	8/14 (57.1)	13/25 (52.0)	32/57 (56.1)	0.93
D8	37/63 (58.7)	4/11 (36.4)	14/20 (70.0)	19/32 (59.4)	0.19
*C. parapsilosis*	109/355 (30.7)	27/81 (33.3)	28/90 (31.1)	54/184 (29.3)	0.807
D1	71/196 (36.2)	22/56 (39.3)	20/45 (44.4)	29/95 (30.5)	0.237
D5	25/96 (26.0)	3/14 (21.4)	5/25 (20.0)	17/57 (29.8)	0.591
D8	13/63 (20.6)	2/11 (18.2)	3/20 (15.0)	8/32 (25.0)	0.67
*Nakaseomyces glabratus*					
(formely *C. glabrata*)	36/355 (10.1)	9/81 (11.1)	11/90 (12.2)	16/184 (8.7)	0.627
D1	13/196 (6.6)	3/56 (5.4)	2/45 (4.4)	8/95 (8.4)	0.611
D5	14/96 (14.6)	2/14 (14.3)	6/25 (24.0)	6/57 (10.5)	0.282
D8	9/63 (14.3)	4/11 (36.4)	3/20 (15.0)	2/32 (6.3)	0.048
*C. tropicalis*	15/355 (4.2)	3/81 (3.7)	4/90 (4.4)	8/184 (4.3)	0.965
D1	11/184 (6.0)	1/56 (1.8)	3/45 (6.7)	7/95 (7.4)	0.334
D5	3/96 (3.1)	1/14 (7.1)	1/25 (4.0)	1/57 (1.8)	0.559
D8	1/63 (1.6)	1/11 (9.1)	0/20 (0.0)	0/32 (0.0)	0.091
*Clavispora lusitaniae*					
(formely *C. lusitaniae*)	4/355 (1.1)	2/81 (2.5)	0/90 (0.0)	2/184 (1.1)	0.311
D1	2/196 (1.0)	2/56 (3.6)	0/45 (0.0)	0/95 (0.0)	0.08
D5	0/96 (0.0)	0/14 (0.0)	0/25 (0.0)	0/57 (0.0)	NA
D8	2/63 (3.2)	0/11 (0.0)	0/20 (0.0)	2/32 (6.3)	0.368
*Meyerozyma guilliermondii*					
(formely *C. guilliermondii*)	3/355 (0.8)	1/81 (1.2)	0/90 (0.0)	2/184 (1.1)	0.594
D1	2/196 (1.0)	1/56 (1.8)	0/42 (0.0)	1/86 (1.2)	0.674
D5	0/96 (0.0)	0/14 (0.0)	0/25 (0.0)	0/57 (0.0)	NA
D8	1/63 (1.6)	0/11 (0.0)	0/20 (0.0)	1/32 (3.1)	0.611
*C. orthopsilosis*	2/355 (0.6)	0/81 (0.0)	1/90 (1.1)	1/184 (0.5)	0.624
D1	2/196 (1.0)	0/56 (0.0)	1/45 (2.2)	1/95 (1.1)	0.543
D5	0/96 (0.0)	0/14 (0.0)	0/25 (0.0)	0/57 (0.0)	NA
D8	0/63 (0.0)	0/11 (0.0)	0/20 (0.0)	0/32 (0.0)	NA
*C. metapsilosis*	1/355 (0.3)	0/81 (80.0)	0/90 (0.0)	1/184 (0.5)	0.628
D1	0/196 (0.0)	0/56 (0.0)	0/20 (0.0)	0/54 (0.0)	NA
D5	1/96 (1.0)	0/14 (0.0)	0/25 (0.0)	1/57 (1.8)	0.708
D8	0/63 (0.0)	0/11 (0.0)	0/20 (0.0)	0/32 (0.0)	NA

^1^Data is presented in numbers (%), unless otherwise stated. ^2^ Not applicable

## Discussion

Our study was designed as an observational, descriptive analysis of fungal colonisation patterns in ICU patients undergoing routine bathing with either 2% CHG-impregnated wipes or 4% CHG liquid soap. Rather than testing the efficacy of CHG, the primary objective was to characterise the prevalence, dynamics, and species distribution of skin fungal colonisation under these two standard-of-care protocols. Given the absence of a non-CHG control group and the observational nature of the design, this study cannot assess the causal impact of CHG on colonisation rates. Instead, our findings provide context-specific insights into fungal colonisation in critically ill patients and highlight temporal trends that may inform future hypothesis-driven or interventional studies. Within this framework, we focused on *C. auris* and *C. parapsilosis* due to their clinical relevance, multidrug resistance profiles, and potential for nosocomial transmission. By sampling patients at ICU admission and during their first week (average stay: ~8 days), we aimed to evaluate any potential associations with CHG bathing practices as applied in routine clinical care.

Skin colonisation poses a substantial risk for hospital transmission and the development of invasive fungal infections, prompting significant interest in exploring effective decolonisation methods [26]. As fundamental components of patient care in ICUs, basic hygiene and skin care have become increasingly important and demand a more rigorous evaluation of antiseptics, with a particular focus on chlorhexidine [27]. Bathing patients with CHG solution is a common way of cleaning patients’ skin in many clinical settings, including intensive care units [28].

The use of chlorhexidine has been evaluated as a potential strategy to reduce the incidence of HAIs, highlighting its importance in infection control protocols [29]. Previous studies have shown that in patients admitted to the ICU who are bathed daily with CHG, a clear inverse relationship was observed between the CHG concentration on the skin and the microbial density [30]. This suggests that higher concentrations of CHG correspond to lower levels of microbial presence, indicating effective microbial reduction [30]. In this context, we evaluated the daily CHG impact by determining the concentration of viable *Candida* spp. in patient swab suspensions.

Our findings showed a progressive increase in colonisation during the ICU stay, with significant differences between the CHG and non-CHG bathing periods. In the full cohort of 675 patients, colonisation was observed in 27.3% on Day 1 (D1), 42.8% on Day 5 (D5), and 52.7% on Day 8 (D8), indicating an overall upward trend despite antiseptic bathing. Longitudinal follow-up of a subgroup of 89 patients—sampled across all three time points—offered additional insights. A modest decline in colonisation was observed from 34.5% on D1 to 30.9% on D5 during the CHG period, followed by a significant increase to 52.7% by D8 (*p* = 0.005). This pattern suggests a possible rebound in colonisation after cessation of CHG use, reinforcing the observation that CHG’s impact may be transient and insufficient for sustained fungal suppression. Although these temporal shifts do not establish causality, they suggest potential differences in colonisation dynamics that merit further study.

These results suggest that *Candida* acquisition continued during the ICU stay, highlighting the need for additional infection control measures. The reasons for this seem to be multifactorial. Routine baths may not adequately distribute CHG to all colonised sites, potentially limiting its effectiveness against *Candida* colonisation. This contrasts with previous findings where CHG successfully reduced skin colonisation by multidrug-resistant Gram-negative and Gram-positive bacteria [6, 29].

One possible reason is the action mechanism of this biocide which acts by destabilising the cell walls of Gram positive and Gram-negative bacteria [27]. For yeast-like fungi, the cell wall is primarily composed of mannans, glucans, and chitin, although the exact arrangement and proportion of these components can vary significantly among different fungal genera associated with major infections [31]. This variability in cell wall composition may influence the effectiveness of CHG baths, as the structural differences could affect how well CHG can penetrate and act against different fungi. Therefore, understanding the specific cell wall biology of these fungi is crucial when considering CHG-based interventions in the ICU.

It is important to highlight that most of the studies do not address the impact of CHG baths especially on microorganisms such as *Candida* species. Instead, they focus on evaluating the effectiveness of daily CHG bathing in reducing nosocomial infections among critically ill patients [6, 8, 29].

Our analysis also explored the impact of different CHG application methods and concentrations, and we did not observe significant differences in *Candida* colonisation throughout the length of stay between ICUs using 2% CHG wipes and one using 4% CHG liquid soap. Nevertheless, previous studies have reported different findings. A meta-analysis by Peixoto et al. revealed that bed bathing with wipes impregnated with 2% CHG reduced the risk of preventing central line-associated bloodstream infections by 48% compared with conventional bed bathing (risk ratio 0.52; 95% confidence interval, 0.37–0.73) [10]. Rhee et al. also reported higher CHG concentrations on the skin when using impregnated wipes, particularly near catheter insertion sites [32]. However, these studies did not evaluate the impact of CHG on viable fungal populations.

When comparing the mycobiota present during the periods when CHG baths were administered to those when conventional soap baths were used (from D6 to D8), there was a slight increase in colony counts, although this increase was not statistically significant. The CFU estimates for these time points did not show significant variations overall, suggesting that the type of bathing solution did not markedly impact the fungal burden. Nonetheless, studies evaluating the impact of CHG versus conventional soap baths in randomized trials over HAI have shown that bathing with 2% and 4% CHG significantly reduces the incidence of HAI in intensive care settings [28, 33]. It is important to note that HAI under evaluation do not include invasive fungal infections. In fact, studies on the effectiveness of CHG on nosocomial fungal infections are lacking.

However, an exception was observed with *C. glabrata*, where a notable decrease in CFUs was recorded on day 8 under the standard bathing practice using regular soap and water soap protocol. This decrease suggests that CHG may have a less pronounced antifungal effect on *C. glabrata* than on other *Candida* species or that *C. glabrata* is more sensitive to the antifungal properties of soap. This finding highlights a potentially unique interaction between CHG and *C. glabrata*, warranting further investigation into the mechanisms underlying this response. This finding also suggests that the effectiveness of CHG as an antifungal agent might vary depending on the specific fungal species present, which could have implications for tailoring infection control protocols in the ICU.

Our findings align with those of a study by Popovich et al., which demonstrated a slight decrease in the mean log10 CFUs of *Candida* 23 h post-bath compared with pre-bath levels [30]. However, other studies have demonstrated the effectiveness of CHG on *Candida* species [34, 35]. Importantly, these studies assessed the density of oral colonisation rather than skin colonisation, and they specifically demonstrated the effectiveness of CHG in reducing the biovolume of viable *Candida* cells in vitro. In vitro assays have shown that yeast-like fungal isolates, including *C. auris*, are inhibited by CHG at concentrations as low as 0.02% (v/v) [11, 36]. Therefore, direct comparison with our study, which focused on skin colonisation, is not feasible.

Importantly, despite global concerns about increasing antifungal resistance among *Candida* species [37], the viable isolates recovered under CHG bathing conditions in our study were largely susceptible to antifungal agents. In our previously published work, we reported a very low overall resistance rate, with only 2.7% of isolates resistant to fluconazole and 99.2% remaining susceptible to voriconazole [38]. Nevertheless, we observed low fungal mycobiota diversity, consisting only of yeast-like fungi and an absence of isolates of the fungal genus potentially described as the main colonisers of human skin, the *Malassezia* genus [39]. A decrease in species diversity associated with a reduction in the number of mixed cultures was also observed throughout the collection period.

Previous research has shown that CHG concentrations on the skin can vary significantly depending on the body site, with the neck typically exhibiting the lowest concentrations [10]. Such variability in CHG distribution could explain its reduced antimicrobial effectiveness in body regions where suboptimal concentrations are achieved. According to Rhee et al. (2023), the axillary and inguinal regions tend to retain higher concentrations of CHG compared to other areas like the neck, which may contribute to more effective antifungal action in these regions [32]. These areas enhance CHG retention due to factors such as increased skin folds, higher moisture levels, or reduced exposure to environmental factors, and lower rates of natural cleansing [30, 32]. Although our study did not sample multiple body sites, it is plausible that higher fungal densities persist in areas where CHG retention is lower, warranting further targeted investigation. Further studies should include a broader range of sampling sites to fully understand the distribution and efficacy of CHG across different body regions, which could inform more targeted and effective infection control protocols.

This study has some limitations that should be considered when interpreting the results. First, differences in CHG application methods (e.g., concentrations and protocols) between the two participating hospitals may restrict the generalizability of the findings. Direct comparisons between 4% and 2% CHG bathing should be interpreted with caution, as these interventions were implemented at two separate institutions with differing ICU profiles and baseline colonization rates, introducing potential confounding factors that may have influenced outcomes independently of CHG concentration.

Second, the reduced sample size—675 enrolled instead of the planned 875, with only 224 patients undergoing follow-up sampling—limited the study’s statistical power to detect colonization differences over time or between intervention groups and should be considered when interpreting the findings. This limitation is further compounded by methodological constraints: all patients were assessed only after their first CHG bath, with no baseline cultures or untreated control samples for comparison, which restricts the ability to establish true pre-intervention microbial profiles. Additionally, the study design included an asymmetrical bathing protocol: CHG was administered for five consecutive days (D1–D5), whereas soap-and-water bathing was applied over three days (D6–D8). This discrepancy in exposure periods complicates direct comparisons of efficacy between the two interventions. Future studies should adopt randomized designs with balanced exposure periods to better assess the specific impact of CHG bathing, especially on multidrug-resistant *Candida* species.

## Conclusions

Our study demonstrated that CHG bathing did not effectively reduce the acquisition or decolonisation of *Candida* spp. among ICU patients. Although CHG is widely used for infection control, its routine application appears to have limited efficacy against fungal colonisation, particularly for skin-resident *Candida* species. These findings suggest that current CHG-based hygiene protocols may need to be supplemented with additional or alternative antifungal strategies, especially given the rising concern over nosocomial fungal infections. Continuous monitoring of fungal burden could serve as a useful tool to evaluate the consistency of skin hygiene practices, even if direct antifungal effects are limited. Furthermore, species-specific responses, such as the unique behaviour of *C. glabrata* observed in our study, highlight the need for tailored decolonisation approaches. Future research should prioritize randomized controlled trials with standardized exposure periods, broader sampling across multiple body sites, and a focused evaluation of CHG’s efficacy against multidrug-resistant *Candida* species to better inform ICU infection prevention protocols.

## Data Availability

No datasets were generated or analysed during the current study.
